# Excess respiratory, circulatory, neoplasm, and other mortality rates during the Covid-19 pandemic in the EU and their implications

**DOI:** 10.1017/S0950268825100265

**Published:** 2025-07-14

**Authors:** Gabrielle Elizabeth Kelly, Stefano Petti, Norman Noah

**Affiliations:** 1 School of Mathematics and Statistics, University College Dublin, Dublin, Ireland; 2 Department of Public Health and Infectious Diseases, Sapienza University, Rome, Italy; 3 Department of Infectious Disease Epidemiology, London School of Hygiene and Tropical Medicine, London, UK

**Keywords:** Covid-19, Excess mortality, Respiratory diseases, Cardiovascular diseases, Neoplasms, Traffic accidents, Public health measures, EU, Central Eastern European Countries, Ireland

## Abstract

This study analyzed standardized excess mortality due to specific causes during the Covid-19 pandemic across 33 European countries, using Eurostat data (2016–2021) and Our World in Data databases. Causes included circulatory and respiratory diseases, neoplasms, transport accidents, and “other” causes (e.g., diabetes, dementia, ill-defined conditions). Additional variables such as vaccination rates, economic and health indicators, demographics, and government stringency measures were also examined. Key findings include: (1) Most European countries (excluding Central and Eastern Europe), recorded lower than expected excess mortality from circulatory and respiratory diseases, neoplasms, and transport accidents. Ireland had the lowest excess respiratory mortality in both 2020 and 2021; (2) Croatia, Cyprus, Malta, and Turkey showed significant positive excess mortality from “other” causes, potentially linked to public health restrictions, with Turkey as an exception; (3) Regression analysis found that higher human development index and vaccination rates were associated with lower excess mortality. Policy Implications are: (1) Statistically significant positive or negative cause-specific excess mortality may indicate future health trends; (2) The pandemic and government stringency measures negatively affected mortality from “other” causes; (3) Strengthening health system resilience, investing in digital medicine, directing aid to countries with weaker systems, and supporting disadvantaged groups are key recommendations.

## Introduction

This article is motivated by the fact that while it has been reported that a substantial increase in excess mortality largely coincided with a Covid-19 outbreak in each European country, the indicator did not specify the causes of death [1–2]. In a recently published article [3], excess mortality rates in 2020 for a snapshot of 35 countries were examined. Residual mortality rates (RMR) (i.e. non-Covid-19 excess mortality) in 2020 were then calculated as excess mortality minus reported Covid-19 mortality rates. Differences in RMR are differences not attributed to reported Covid-19, and in 35 countries, about half the RMR’s were negative.

The first aim of this article was therefore to examine excess mortality rates due to non-Covid-19 causes and to provide insights into its main causes of death, separately for each country and for both 2020 and 2021. Circulatory diseases accounted for close to one-third (32.4%) of all deaths in the European Union (EU) in 2021. The second most common cause was cancer (21.6%), followed by Covid-19 (10.7%) and respiratory diseases (6.0%) [4]. These non-Covid-19 causes of death are investigated here as being most likely to show the greatest change, together with a possible reduction in deaths from road traffic accidents that did not occur due to restrictions on commuting or travel during the lockdown periods. ‘Other’ (grouped) non-Covid-19 causes are also investigated together with an exploratory analysis of contributory causes to possible changes in this category. Our second aim was to analyze associations between the excess mortality rates and vaccination rates, economic, health, and demographic indicators, and government response stringency index (SI). Thirdly, we highlighted differences between countries in terms of characteristics of their economic, social, and health systems. Much research has focussed on the elderly or those who have pre-existing health conditions as being at high risk of dying of Covid-19, with being male also a factor [5–6]. The prevalence of some health conditions, however, is often country specific and we focussed on this. The results are then used to indicate which preventive and medical curative measures or which investment in research might be used in a future pandemic.

## Methods

Data were obtained from the Eurostat database [7]. From this database, yearly data on causes of death for 32 countries, namely, all 27 EU Member States, European Free Trade Association (EFTA) countries (Iceland, Liechtenstein, Norway, Switzerland), the two candidate countries Serbia and Turkey, were available from 2016–2021 inclusive. Monthly data were unavailable for causes of death prior to 2019, and therefore, attention is restricted here to yearly data. Eurostat statistics on the causes of death are based on the medical information provided in the death certificates. Causes of death are based on the International Classification of Diseases and Related Health Problems (ICD-10) codes, tenth revision. Codes U071 (virus identified, deaths where Covid-19 has been confirmed by laboratory testing), U072 (virus not identified, denoted by CNID), and U_COV19_OTH (Covid-19 death not elsewhere defined) were used to identify whether a death was related to Covid-19 infection codes. Covid-19 identified will be denoted by CID and otherwise by Covidother (U072 + U_COV19_OTH).

Standardized death rates (SDR) are examined rather than crude, as the population structure strongly influences this indicator for broad age classes. Details of their calculation can be found on the Eurostat database [8]. The excess mortality rate 2020 and 2021 is defined and calculated (as by Eurostat) as the SDR from all causes minus the average annual SDR over the previous four years (2016–2019) before the pandemic.

Similarly, we calculated pre-planned excess death rates due to the specific causes: Circulatory diseases (codes I00-I99), neoplasms (codes C00-D48), non-Covid-19 respiratory diseases (codes J00–J99), transport accidents (codes V-Y85), and ‘other’ (non-Covid-19 causes excluding the four previous) for 32 countries in 2020 (Turkish data unavailable) and 33 in 2021. Mortality rates were assumed to follow a Poisson distribution, and standard deviations of excess rates were calculated based on this assumption and then tested for statistical significance using z-scores as in [9–10]. The excess non-Covid-19 mortality rate for each country was calculated as the excess death rate minus death rates due to Covid-19 (identified, non-identified, other) [5].

Another dataset was obtained from the OWID database [11]. Variables provided included the SI as defined below, population size, population density, median age, aged 65 or older, aged 70 or older, per-capita GDP (gdp), extreme poverty, female smokers, male smokers, hospital beds per thousand, life expectancy, and human development index (HDI, a composite index measuring average achievement in three basic dimensions from the United Nations Development Programme: Life expectancy, education, and gross national income per capita). More information is available in the codebooks. The SI proposed in [12] is an indicator of the measures taken by governments against Covid-19. This is a composite metric based on nine reaction indicators, such as school closures, workplace closures, and travel restrictions, rescaled to a number between 0 and 100 (100 being the most stringent). People vaccinated per hundred of the population was available from the database for each day 2020 and 2021. Excess mortality rates for specific causes were only available per year, so to allow for comparisons and correlations, the mean vaccination rate for 2020 and 2021 was calculated for each country, as well as the maximum (max), minimum (min), and standard deviation (sd). The same procedure was carried out for the SI. Correlation and stepwise regression analyses were carried out relating excess mortality rates separately for each cause and for 2020 and 2021 to these explanatory variables. Spearman correlation coefficients were computed in all cases. Statistical significance is denoted by a *p*-value <0.05. The percentage of variation explained by a regression model is denoted by R^2^.

The Organisation for Economic Co-operation and Development (OECD)’s definition of central and eastern European countries (CEECs) for the group of countries comprising Albania, Bulgaria, Croatia, Czechia, Hungary, Poland, Romania, Slovakia, Slovenia, Estonia, Latvia, and Lithuania was used [13].

## Results

Note that results concerning Liechtenstein will not be commented on due to its small population and subgroup sizes, where statistically significant results in rates may only reflect very small differences in absolute numbers. Detailed results on the regression models are shown in the Supplementary Material. As the HDI was statistically significant in most models results for it, together with vaccination statistics and the SI are shown here. Correlations with stringency statistics are noted separately, even if they are not statistically significant. Results relating to CID and Covidother standardized mortality rates are in the Supplementary Material and have been discussed in part elsewhere [1, 11].

### Overall excess and non-Covid-19 excess mortality

**Figure 1.**
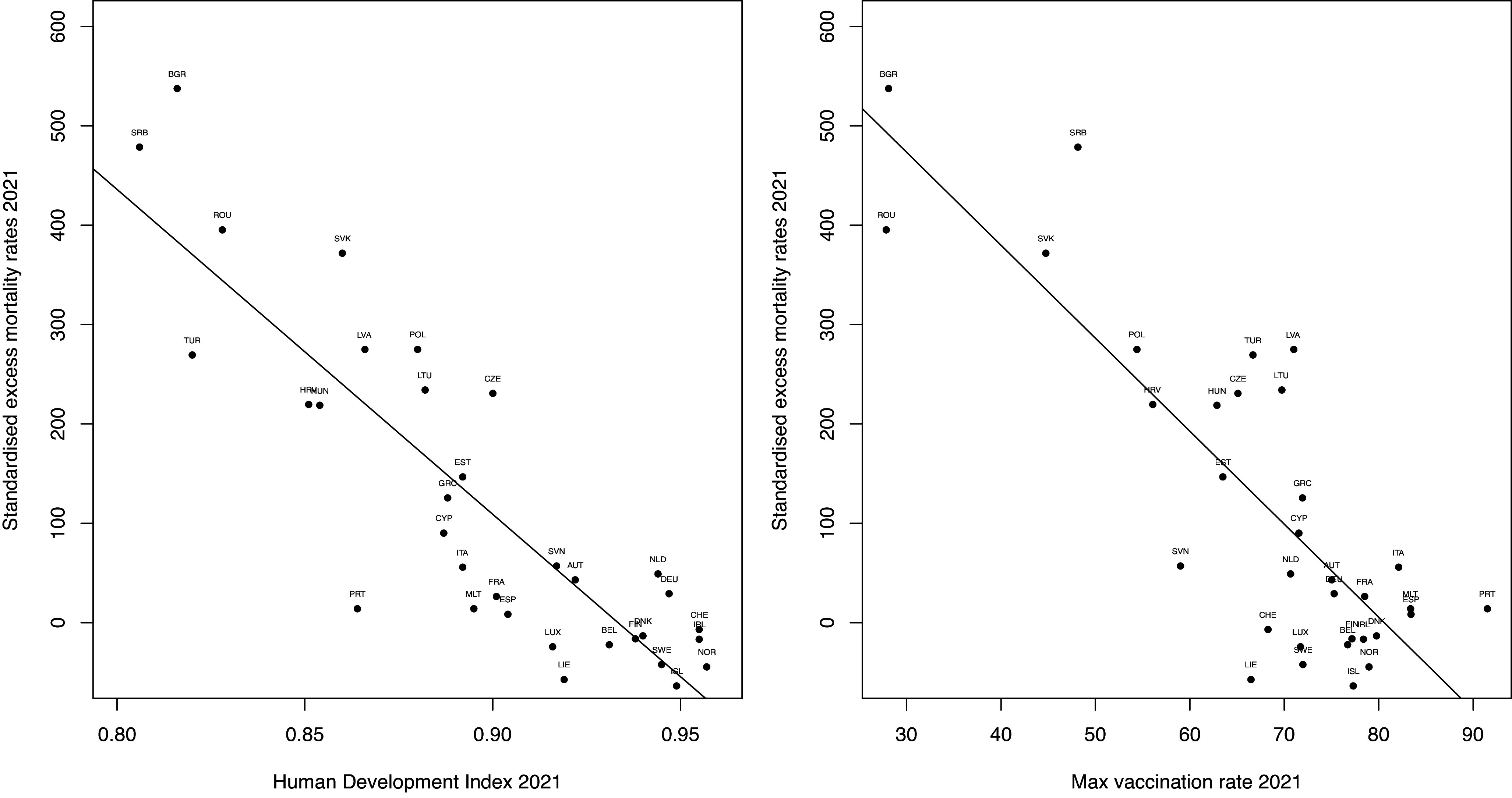
Standardized excess death rate per 100,000 of the population versus the human development index and max vaccination rate per 100 population for 33 countries in 2021 with regression lines. Countries are labelled by their ISO code.

Tables 1 and 2 show that overall excess mortality rates were negative for six countries in 2020 and for nine in 2021 but not significantly so in either year, while most countries were positive, many significantly so. Countries in eastern Europe had the highest excess mortality rates in 2021. Thirteen countries had lower rates in 2021 than in 2020, with many showing marked decreases, while in some CEEC countries, rates more than doubled. In the stepwise regression analysis, in both 2020 and 2021, the HDI (*p* = 0.04, *p* < 0.0001, respectively) was negatively associated with excess mortality, and in 2021, the SI mean (*p* = 0.08) was positively associated and max vaccination rate (*p* = 0.0001) was negatively associated. Figure 1 displays the association between the excess mortality rate and the HDI and vaccination rate in 2021. Seven of 32 countries had positive non-Covid-19 excess for 2020 and eight countries in 2021; these were all CEEC countries apart from Italy in 2020 and Cyprus and Turkey in 2021 (Tables 1 and 2 columns (6) + (7), Supplementary Figure S1).

**Table 1.** Excess standardized mortality rates per 100,000 population overall for specific causes and Covid-19 mortality rates in 32 European countries in 2020^†^

Country	Overall (1) = (6) + (7) + (8)	Circulatory (2)	Neoplasms(3)	Respiratory (4)	Transport (5)	Sum columns (2)–(5) = (6)	‘Other’ non- Covid (7)	Covid-19 (8)
Austria	54.83	−20.49	−2.67	−5.62	−0.87	−29.65	13.09	71.39
Belgium	107.22**	−31.18	−16.02	−18.92	−1.16	−67.28	−6.97	181.48
Bulgaria	192.29***	37.00	10.57	18.21	−2.15	63.63	14.88	113.77
Croatia	73.02	−19.34	−19.84	−11.22	−1.89	−52.29	17.90	107.42
Cyprus	−0.83	−23.07	3.97	−3.23	0.00	−22.33	2.04	19.45
Czechia	141.11***	−3.23	−5.73	−0.81	−0.64	−10.41	41.59*	109.92
Denmark	−35.72	−27.51	−13.85	−18.44	−0.28	−60.08	5.07	19.3
Estonia	−33.30	−49.98	−18.48	−7.31	−0.08	−75.85	28.10	14.45
Finland	−29.02	−28.53	−4.17	−8.13	−0.40	−41.23	3.06	9.16
France	41.50	−19.35	−10.5	−9.40	−1.23	−40.48	−4.76	86.75
Germany	4.19	−22.93	−7.31	−10.1	−0.62	−40.96	4.91	40.23
Greece	28.59	−10.1	−3.90	−12.17	−1.71	−27.88	17.67	38.79
Hungary	79.76	−6.07	−14.87	−11.25	−1.95	−34.14	17.69	96.2
Iceland	−43.33	−21.82	−15.42	−14.59	−0.16	−51.99	−3.24	11.91
Ireland	−38.83	−37.08	−12.11	−34.97**	−0.17	−85.33	−9.44	54.94
Italy	109.89**	−7.02	−9.54	4.75	−1.48	−13.29	22.47	100.72
Latvia	−7.90	−33.88	0.65	−6.647	−0.75	−40.63	−2.06	34.76
Liechtenstn	120.6***	−109.0***	−1.27	−32.45***	1.82	−140.9	131.19	130.32
Lithuania	93.00*	−0.08	3.04	−4.07	−0.53	−1.64	18.43	76.21
Luxembourg	21.30	−46.09**	−28.64	−9.3	−1.04	−85.07	6.82	99.55
Malta	36.21	−31.83	−8.98	−1.28	−1.85	−43.94	36.59*	43.57
Netherlands	57.88	−31.47	−12.71	−19.77*	−0.39	−64.34	−2.53	124.74
Norway	−48.18	−27.24	−12.95	−19.65	−0.66	−60.5	3.59	8.72
Poland	163.82***	−3.33	−7.64	4.17	0.03	−6.77	49.45*	121.14
Portugal	57.81	−2.45	−3.61	−20.41	−1.20	−27.67	26.21	59.27
Romania	156.28***	43.86	−11.88	25.32*	−1.21	56.09	8.16	92.03
Serbia	170.46***	−35.23	−10.69	18.93	−0.91	−27.9	53.13*	145.23
Slovakia	60.97	−0.27	−6.99	10.177	−0.97	1.95	−27.94	86.98
Slovenia	118.51**	−35.39	−12.38	−21.34**	−0.46	−69.57	26.36	161.73
Spain	98.64**	−11.03	−9.96	−18.06	−0.77	−39.82	0.44	138.01
Sweden	42.44	−31.32	−10.55	−12.02	−0.49	−54.38	6.91	89.9
Switzerland	65.44*	−16.32	−14.73	−11.49	−0.52	−43.06	2.55	105.94

† *, **, *** denotes statistical significance at the 0.05, 0.01, and 0.001 level, respectively.

**Table 2.** Excess standardized mortality rates per 100,000 population overall for specific causes and Covid-19 mortality rates in 33 European countries in 2021^†^

Country	Overall (1) = (6) + (7) + (8)	Circulatory(2)	Neoplasms(3)	Respiratory(4)	Transport(5)	Sum columns (2)–(5) = (6)	‘Other’ non-Covid(7)	Covid-19(8)
Austria	42.97	−38.20	−8.83	−15.40	−0.76	−63.19	20.61	85.55
Belgium	−22.29	−32.07	−18.67	−29.74**	−1.06	−102.77	−24.73	83.99
Bulgaria	537.45***	128.29***	−5.40	29.82**	−2.15	150.56	13.34	373.54
Croatia	219.80***	−9.00	−15.63	−4.07	−0.71	−29.41	45.20*	204.02
Cyprus	90.33*	−30.00	12.37	−20.92	−0.74	−39.29	42.46	87.15
Czechia	230.80***	−32.77	−16.02	−7.49	−1.10	−57.38	34.65	253.53
Denmark	−13.25	−23.05	−14.40	−15.61	−0.65	−53.71	14.56	25.91
Estonia	146.88***	−10.79	−18.02	−0.35	0.28	−28.88	49.36**	126.41
Finland	−16.39	−31.35	−6.18	−8.77	−0.25	−46.55	14.78	15.39
France	26.30	−17.11	−14.75	−12.50	−0.87	−45.23	−5.74	77.28
Germany	29.03	−26.15	−12.18	−14.97	−0.77	−54.07	9.97	73.12
Greece	125.51***	−5.72	−6.17	−8.71	−1.38	−21.98	20.29	127.2
Hungary	218.66***	−14.63	−26.60	−15.94	−1.13	−58.3	18.78	258.19
Iceland	−63.68*	−31.84	−21.46	−16.64	−1.18	−71.12	5.75	1.7
Ireland	−16.56	−34.21	−20.37	−40.27***	−0.53	−95.38	−6.41	85.23
Italy	55.73	−21.27	−15.53	−10.46	−0.51	−47.77	20.98	82.52
Latvia	274.90***	42.05	−11.69	−3.46	0.23	27.13	32.39	215.36
Liechtenstein	−57.09	−65.59***	−5.49	−68.81***	−1.57	−141.46	27.28	57.09
Lithuania	234.16***	7.13	−14.66	−9.00	−1.75	−18.28	15.09	237.34
Luxembourg	−24.24	−57.35***	−27.80	−16.92	−1.72	−103.79	−6.43	85.98
Malta	14.01	−46.08*	−22.96	−1.58	−3.18*	−73.8	43.93*	43.89
Netherlands	48.99	−31.29	−17.66	−23.93**	−0.64	−73.52	4.49	118.02
Norway	−44.37	−18.74	−17.29	−23.67*	−1.16	−60.86	−1.14	17.62
Poland	275.19***	14.32	−29.47	1.55	−2.31	−15.91	23.69	267.41
Portugal	14.09	−39.53*	−17.92	−34.43***	−1.04	−92.92	4.20	102.81
Romania	395.52***	131.12***	−29.04	40.12**	−0.96	141.24	31.54	222.74
Serbia	478.45***	38.82	−17.41	28.24*	−0.24	49.41	29.03	400.01
Slovakia	371.90***	33.86	−31.95	73.44***	−1.09	74.26	−19.25	316.9
Slovenia	56.99	−51.30*	−25.99	−28.41***	0.08	−105.62	22.34	140.27
Spain	8.62	−15.88	−11.13	−31.43***	−0.53	−58.97	−6.86	74.44
Sweden	−42.20	−41.57*	−18.63	−17.51*	−0.73	−78.44	−13.42	49.65
Switzerland	−6.88	−30.40	−19.00	−13.82	−0.69	−63.91	−10.08	67.11
Turkey	269.33***	37.12	−22.68	48.78**	−2.50	60.42	51.64*	156.98

† *, **, *** denotes statistical significance at the 0.05, 0.01, and 0.001 level, respectively.

### Cause-specific excess mortality: Respiratory diseases

**Figure 2.**
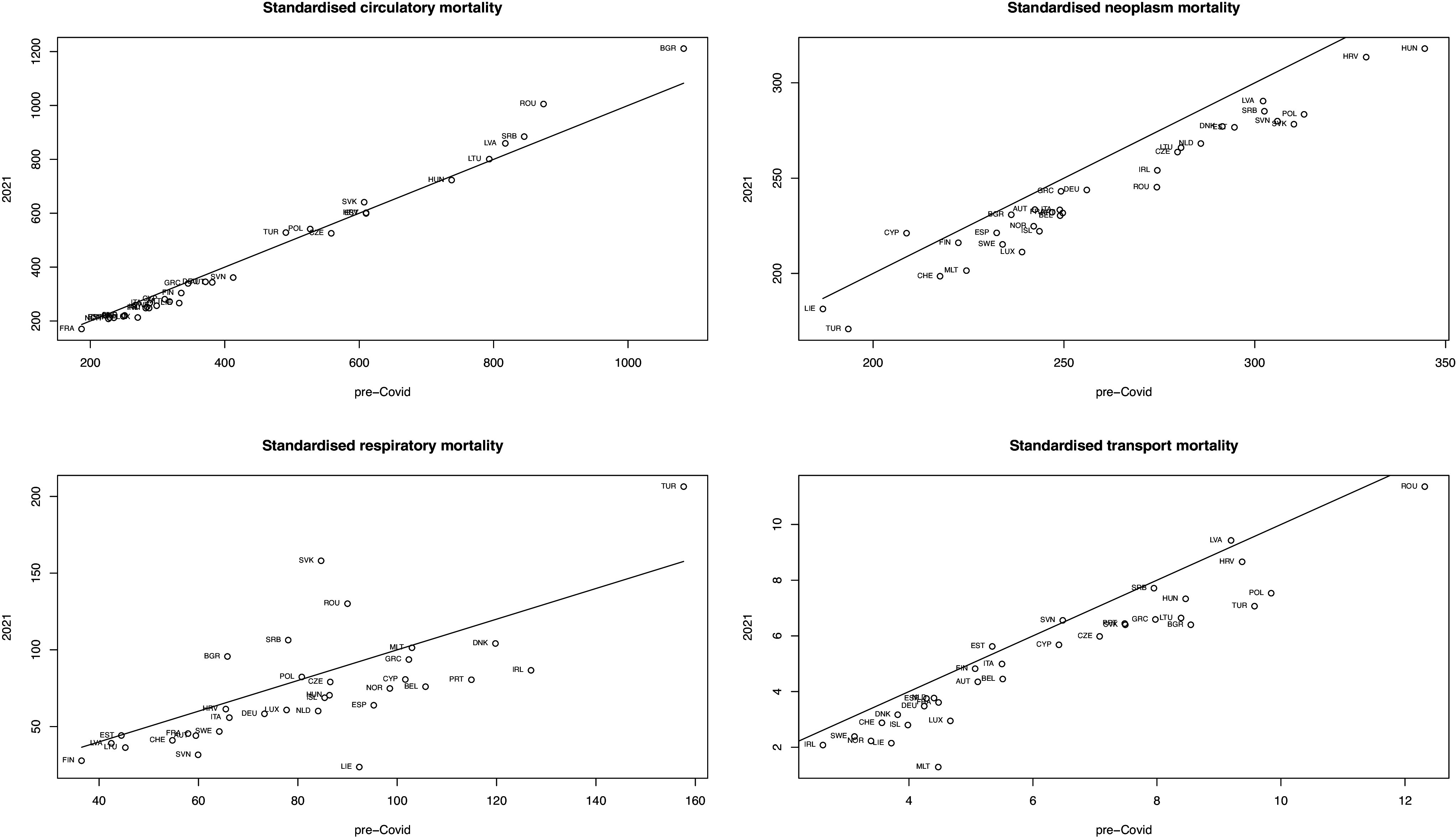
Mortality rates standardized per 100,000 of the population for specific causes pre-Covid and in 2021 with equality lines. Pre-Covid rates are the averages for 2016–2019. Countries are labelled by their ISO code.

Only six countries had positive excess mortality due to respiratory diseases during 2020; these were all eastern European countries bar Italy, and Bulgaria; Romania and Serbia were again positive in 2021 – significantly so (Tables 1 and 2, Figure 2). Two countries had significantly negative excess respiratory mortality in 2020 – Ireland and Slovenia, and ten countries had significantly negative excess respiratory mortality in 2021. Bulgaria, Romania, Serbia, and Slovakia had marked higher excess rates in 2021 than in 2020, while in other countries, rates were comparable or lower. Excess respiratory mortality was negatively related to HDI in 2020 and 2021 (*p* < 0.001, *p* = 0.003, respectively), and in 2021, it was negatively correlated with mean vaccination rate (*r* = −0.61, *p* = 0.0002), sd of vaccination rate (*r* = −0.56, *p* = 0.0008), and max vaccination rate (*r* = −0.54, *p* = 0.0013). It was also negatively correlated with the mean SI in both 2020 and 2021 but not significantly.

### Cause-specific excess mortality: Circulatory diseases

Some CEEC’s had the highest mean circulatory diseases mortality rates in 2016–2019. Luxembourg had significantly negative excess circulatory rates in 2020, while Bulgaria and Romania were the only countries positive. Seven CEEC countries were positive in 2021 with Bulgaria and Romania significantly so (Tables 1 and 2, Figure 2). Rates for 2020 and 2021 were comparable in most countries, apart from several CEEC countries that showed marked increases and Portugal a marked decrease.

In 2020, HDI was negatively (*p* < 0.001) associated with excess circulatory, and in 2021, the mean vaccination rate (*p* < 0.001) and HDI (*p* = 0.003) were negatively associated. Note that excess circulatory mortality was positively correlated with the mean, sd, and max SI in 2020 and positively with the mean and negatively with the sd and max in 2021 but not significantly.

### Cause-specific excess mortality: Neoplasms

Some CEEC’s had the highest mean neoplasms mortality rates 2016–2019. Bulgaria, Cyprus, Latvia, and Lithuania were positive for excess neoplasms in 2020; all other countries were negative, while in 2021, all countries except Cyprus were negative (Tables 1 and 2, Figure 2). No country was significant in either year. Rates for 2021 were somewhat lower in all countries than in 2020. In 2021 HDI was positively associated with excess neoplasms (p = 0.005). Excess neoplasms in 2021 were negatively correlated with min vaccination rate (*r* = −0.37, *p* = 0.036). Correlation coefficients between excess neoplasms in 2020 with the SI in 2020 and 2021 statistics were all very small and negative.

### Cause-specific excess mortality: Transport accidents

Excess rates due to transport were almost all negative in 2020 (except Poland) and 2021 (except Estonia, Latvia, and Slovenia, Figure 2) although none significantly so, and rates were relatively low. There was little change in 2021 from 2020. Excess transport was positively associated with HDI in 2020 and 2021 (*p* < 0.001, *p* = 0.0194, respectively). Excess transport in 2021 was not significantly correlated with vaccination statistics, and all correlations were very small. All correlations of excess transport with SI statistics were negative but not significant in 2020 and 2021. Excess transport in 2021 was not significantly correlated with SI 2020 statistics.

### Cause-specific excess mortality: ‘Other’ non-Covid-19

Excess rates due to ‘other’ causes were almost all positive in 2020 and 2021 although few significantly so (Tables 1 and 2, Supplementary Figure S1). Croatia, Cyprus, Estonia, Latvia, and Romania showed marked increases from 2020 to 2021, while Belgium, Poland, and Portugal marked decreases. In 2021, ‘other’ non-Covid-19 excess was significant and almost equal to the sum of the four in Turkey, with significant positive excess in certain infectious and parasitic diseases (ICD-10: A00–B99) and in ill-defined and unknown causes of mortality (ICD-10: R96–R99) (*p* < 0.001). Croatia had significant ‘other’ mortality in 2021 due to highly significant positive rates of excess diabetes mortality in both 2020 and 2021 (*p* < 0.001), while though in most countries rates were positive, they were low. Malta also had significant ‘other’ mortality in both 2020 and 2021 and is the only country to have significant positive excess mortality due to dementia (ICD-10: F01–F03) both years with rates almost double the next largest country. In other countries, some excess dementia rates were positive and some negative. Czechia, Poland, and Serbia had significant ‘other’ excess mortality in 2020 and Estonia in 2021 due to small increases in a number of different mortality rate causes.

In 2021, excess ‘other’ was negatively associated with HDI (*p* = 0.014) and positively associated with excess respiratory in 2021 (*p* = 0.005). Excess ‘other’ was positively correlated with maximum vaccination rate in 2021 (*p* = 0.037), but the correlation was small. Excess ‘other’ in 2021 was not significantly correlated with SI or vaccination statistics in 2020. All correlations of excess ‘other’ with SI statistics were not significant in 2020 and 2021.

## Discussion

This is a novel study that examines excess death rates due to the leading mortality causes in European countries during the pandemic years of 2020 and 2021. Other studies have examined smaller groups of countries and not necessarily in Europe [6, 9, 14]. Each of the cause-specific excess mortality rates is discussed separately in what follows.

### Overall excess and Covid-19 mortality

Overall excess and Covid-19 mortality in the EU has been extensively examined (e.g. [1]) and will be briefly discussed here.

For most countries, excess mortality rates were positive in both 2020 and 2021. However, non-Covid-19 excess mortality rates (Tables 1 and 2, columns (6) + (7), Supplementary Figure S1), were typically negative outside of the CEEC region. This means that the number of reported Covid-19 deaths is greater than the number of non-Covid-19 excess mortalities and that non-Covid-19 deaths have been lower than expected during the pandemic. For example, Italy had a positive non-Covid-19 excess in 2020. One possible explanation is that Italy reported its first Covid-19 death early (22 February 2020), prior to WHO’s April 2020 guidance, that Covid-19 should be listed as the underlying cause whenever it was confirmed or suspected. Italy had its highest respiratory monthly mortality rate of 12.71, for 2019–2020, in March 2020, with the next highest month being just 8.3. The remainder 9 months in 2020 were comparable to 2019. Consequently, some early deaths in Italy may have been attributed to respiratory causes, artificially inflating non-COVID excess mortality. It is worth noting again that monthly excess mortality in 2020 could not be precisely computed as Eurostat monthly data are only available from 2019 onwards.

Consistent with [9], higher vaccination rates were associated with lower excess mortality. Stringency variables and vaccination rate variables were all positively correlated in 2021, indicating that countries with responsible public health policies had both high vaccination and stringency rates. The SI was not associated with excess mortality rates, perhaps indicating that restrictions were adapted in most countries to older and vulnerable populations.

Most countries experienced higher rates of Covid-19 mortality in 2021 than 2020 as the pandemic spread. It is stated in [15], Covid-19 mortality can be estimated from excess deaths only when reliable mortality data are routinely available. However, this is difficult even then. Data on Covid-19 mortality and other reports show that only 1%–3% of all Covid-19 deaths were free of comorbidities [15–18]. Deaths attributed to Covid-19 cannot be attributed to other causes; consequently, rates of other causes of death may be reduced. This may have happened in Belgium that had a high rate of both CID and Covidother mortality (Supplementary Table S1) and low non-Covid-19 excess (Tables 1 and 2). Belgium included deaths in care homes that were suspected but not confirmed as Covid-19 cases. Belgium also has a high rate of care home occupancy relative to its population [3]. This supports the hypothesis that if Covid-19 is over-reported, non-Covid-19 excess will be negative.

The proportion of female smokers was positively associated with CID. This was also observed in [3], who linked it to countries with fewer resources to deal with the pandemic. Vaccination rates were negatively associated with CID mortality in 2021 as in [9] and marginally with ‘Covid-19 other deaths’, as expected.

### Cause-specific excess mortality: Respiratory and circulatory diseases

For both circulatory and respiratory diseases, remarkably many CEEC countries recorded their highest monthly rates for 2019–2020 in the last two months of 2020, indicating seasonal effects and other factors.

Bulgaria and Romania, in particular, had notably high excess mortality in 2020 and 2021, with significant contributions from circulatory and respiratory causes. This likely reflects pre-existing health burdens rather than underreporting of Covid-19, as both had high confirmed Covid-19 mortality rates and relatively low ‘Covid-19-other’ rates (Supplementary Table S1). These countries already had the highest circulatory disease rates in the EU prior to the pandemic. It is reported they, together with Croatia, all entered the Covid-19 crisis with common problems, including workforce shortages and underdeveloped and underutilized preventive and primary care, and these compounded the pandemic’s impact. Like other European countries, non-Covid-19 healthcare services were disrupted [19]. Moreover, delays in seeking health services can increase the chance of spreading the virus to others and can result in more severe cases presenting to a healthcare facility – hence the high rates of Covid-19 in these countries.

Negative excess mortality in seven/eight of 12 countries for respiratory and pneumonia in both 2020/2021, and for six of the nine countries, excess cardiovascular mortality in 2020 and 2021 was found in [9]. These results are in broad agreement with those here. Note that countries with negative total non-Covid-19 excess in 2021 are precisely the countries that have statistically significant negative excess respiratory mortality in 2021 likely due to reduced influenza transmission [20].

Regression models showed that the HDI was a significant negative predictor of excess respiratory and circulatory deaths. This highlights HDI as a strong indicator of pandemic preparedness. Median age was negatively associated with excess respiratory mortality, as expected as it affects mostly older age groups. Vaccination rates were negatively associated with respiratory and circulatory excess mortality as in [9], as expected.

### Cause-specific excess mortality: Neoplasms

Comparison of monthly data for 2019 with 2020 showed no neoplasm mortality differences for any country. Positive cancer excess mortality in 8/9 countries in 2020 and 6/9 in 2021 was reported in [9], though only four showed significant increases. For the three countries in common with this study – Austria (negative), Cyprus (positive), and Slovenia (negative) in 2021, the results agree. Differences with [9] can be attributed to different healthcare systems in different countries and the use of standardized rates in this study.

Cyprus in both 2020 and 2021 and Bulgaria, Latvia, and Lithuania in 2020 had positive excess neoplasms mortality rates, while it was negative or else near zero in all other countries (Tables 1 and 2, Figure 2). Cyprus had the highest excess neoplasm rate among countries in this study. Given its low Covid-19-other rate (Supplementary Table S1), the excess is likely due to pre-existing health system weaknesses [21]. Cyprus also had the highest male smoking rate in the study, with Greece and Latvia close behind. Lockdowns may have further increased smoking rates. Similarly, the proportion of female smokers was positively associated with neoplasm excess in 2021 (Supplementary Material). The proportion of the population aged 70 and older was also positively associated (Supplementary Material), reflecting the age-related nature of cancer.

Interestingly, circulatory and neoplasm mortality – the two leading causes of death in Europe – were generally lower than expected in most countries except CEECs. This may be due to these negative excess deaths being classified as ‘comorbidities’ or underlying causes of death in death certificates. However, we have no data to confirm this.

### Cause-specific excess mortality: Transport accidents

As expected, excess mortality due to transport was negative in all countries except Poland in 2020 and Estonia, Latvia, and Slovenia in 2021. These findings likely reflect reduced travel due to lockdowns. Estonia’s outlier status may be linked to its having the lowest mean SI in both years. HDI was positively associated with excess transport mortality, possibly due to greater car ownership and road use in wealthier countries. Although CEECs had the highest transport accident rates, pre Covid-19, strict lockdowns may have reduced fatalities. The proportion aged 70+ was positively associated with excess transport mortality as expected, but since rates are low, the effect is minimal.

### Cause-specific excess mortality: ‘Other’ non-Covid-19

Excesses in circulatory and respiratory diseases, neoplasms, and transport accounted for most of the non-Covid-19 excess, but a significant ‘other’ component remained in some countries in both years. Turkey, for example, had the highest rate of ill-defined and unknown cause of mortality in 2021 and just behind Czechia and Serbia in 2020, while in most countries, rates were positive but low in both years. Croatia, which already had high endocrine mortality rates pre-pandemic, experienced excess diabetes mortality, likely exacerbated by lifestyle factors such as poor diet, smoking, alcohol use, and reduced physical activity [22]. Hypothetically, these factors may have been exacerbated by government stringency measures.

Cyprus also had a high endocrine mortality rate pre-pandemic and experienced an increased rate in 2021. Malta had significant ‘other’ excess mortality in both years. The major causes of disease in Malta are circulatory, respiratory, and then Alzheimer’s and other dementias [23]. While Alzheimer’s mortality declined slightly, dementia-related mortality did not, possibly due to stress related to lockdowns in this densely populated country. In Belgium, Ireland, and France, ‘other’ non-Covid-19 excess mortality (column (7) Tables 1 and 2) was negative in both years likely due to Covid-19 over-reporting in the case of Belgium in particular and decreased mortality due to other causes.

### Policy implications and recommendations

Among the four primary causes of death studied, excess respiratory diseases stand out as having the greatest number of significantly negative excess mortality results, especially in 2021. This appears linked to the SI, a result also found by [9]. This suggests the adoption of face masks and similar pandemic measures during a flu season might be worthwhile [24–25].

However, there are caveats. For example, a May 2024 childhood pneumonia outbreak in China was linked to weakened immunity caused by prior isolation, a pattern also observed in the UK and the USA in 2022 [26]. In Ireland, which has the highest respiratory mortality rates pre-Covid-19 in the EU and the lowest pandemic excess, excess pneumonia and influenza mortality was reported over four consecutive weeks in 2022/2023 [27]. This may reflect a survival effect or weakened immunity. Therefore, there is concern that stringency measures can have both positive and negative effects. Studies in England and Wales have shown a substantial reduction in presentations to hospitals with acute cardiovascular conditions during the pandemic [20, 28]. The pandemic also disrupted cancer treatment access [29–30]. The maintenance of healthcare for severe illnesses during a pandemic is a challenge as hospitals struggle to care for surges of infections.

Continued investment in healthcare system resilience is needed particularly for CEEC countries that experienced Covid-19 and cause-specific excess mortality increases in 2021. The expansion of digital network medicine (i.e. telemedicine and mobile health apps) so that patients continue to receive appropriate care without risking exposure to contagions may have a role in this [31].

Although this study found limited evidence of government policy effectiveness, social control measures still play a role in pandemic response [32–33]. Policies must also target disadvantaged groups, especially those suffering from mental health burdens [34].

It is clear from the results on CEECs including Croatia, Cyprus, Malta, Turkey [35], and Ireland that the pandemic affected non-Covid-19 mortality in different ways and indicates the need for country-specific strategies. A significant shift, positive or negative in any cause of death, is a cause for concern and could signal future risk. Preparing for a future pandemic will require solutions to health problems particular to each country as well as tackling the social determinants of health. For CEECs, relatively low vaccination rates are a problem, and the HDI remains a critical indicator.

As the IMF [36] noted, ‘internationally, strong multilateral cooperation is essential to overcome the effects of the pandemic, including to help financially constrained countries facing twin health and funding shocks, and for channelling aid to countries with weak healthcare systems’. Positively, with the establishment of the EU’s Health Emergency Preparedness and Response Authority (HERA) in 2021, the EU will be better prepared for a future pandemic and avoid past mistakes [37].

## Limitations

Note that in this study, using the average numbers of deaths from past years might underestimate the total expected numbers because of population growth or ageing, but this is unlikely to affect results in any major way as the average is based on just four past years.

Examination of specific causes of excess mortality (beyond the five planned) in the Results section in ‘other’ non-Covid excess mortality was exploratory. Consequently, the associated *p*-values should be interpreted with caution.

Monthly data were only available for specific causes on the Eurostat database starting in 2019, so monthly excess rates could not be computed.

## Supporting information

10.1017/S0950268825100265.sm001 Kelly et al. supplementary material Kelly et al. supplementary material

## Data Availability

The data that support the findings of this study are openly available in the sources listed in the Methods section above.

## Supplementary material

The supplementary material for this article can be found at http://doi.org/10.1017/S0950268825100265.
