# Supplementary material for: Excess respiratory, circulatory, neoplasm, and other mortality rates during the Covid-19 pandemic in the EU and their implications
Source: Epidemiol Infect. 2025 Jul 14;153:e86. doi: 10.1017/S0950268825100265 (PMC12322785; doi:10.1017/S0950268825100265)
Supplement: Kelly et al. supplementary material [file S0950268825100265sup001.zip › GKelly-Revision-Supplement_NTC.docx]

**Excess respiratory, circulatory, neoplasm and other mortality rates during the Covid-19 pandemic in the EU and their implications**

Gabrielle Kelly, Stefano Petti and Norman Noah.

Supplementary Material

Methods.

In the regression analyses univariate regression was initially carried out and variables significant at the 10% level were entered into a stepwise regression analysis. The reduced model included only those variables significant at the 5% level. Since HDI was significantly positively correlated with vaccination statistics in 2021, the latter often did not appear in the final regression model and relevant correlations are noted separately. Correlations (Spearman) with stringency statistics are also noted separately even if they are not statistically significant.

Results.

Part I. Results relating to Covid-19 identified (CID) and Covid-19 non-identified plus other (Covidother) standardised mortality rates.

Firstly, note that most countries had higher rates of Covid-19 mortality in 2021 than in 2020 but Belgium, Italy, Spain, Sweden and Switzerland showing marked decreases. All the CEEC countries showed marked increases apart from Slovenia, with rates more than doubling in some countries.

Covid-19 identified (CID).

From Table S1, Belgium and Slovenia have the highest rates of CID in 2020, while it is the CEEC’s countries (apart from Turkey) that have the highest rates in 2021. CID was positively correlated with excess respiratory 2021 (r=0.54, p=0.0012), excess circulatory 2021 (r=0.61, p=0.0002), excess other 2021 (r=0.36, p=0.0410) but not with excess neoplasms or excess transport, in agreement with the results of [1]. In a stepwise regression analysis in 2020, sd SI was positively (p=0.0413) associated with CID (R^2^=0.14), and in 2021, female smokers (p<0.005) and max SI 2021 (p=0.0916) were positively associated while max vaccination rate (p=0.0097) and life expectancy (p<0.0001) were negatively associated (R^2^=0.86). CID in 2021 was negatively correlated with mean vaccination rate (r=-0.69, p<0.0001), sd vaccination rate (r=-0.77, p<0.0001) and max vaccination rate (r=-0.75, p<0.0001). CID was not correlated with stringency statistics in either 2020 or 2021 though all were positive. CID in 2021 was correlated with sd SI 2020 (r=0.45, p=0.0093) and with max SI 2020 (r=0.38, p=0.0272).

Covid-19 non-identified plus other (Covidother)

Belgium had an extremely high rate of Covidother in 2020 in comparison to other countries and it is again comparatively high in 2021 (Table S1). Serbia also had high rates for both years but in general rates are low for all countries in comparison to CID rates. Most countries had decreased rates in 2021 with just Czechia and Poland showing notable increases from 2020. Covidother was not correlated with excess respiratory, excess circulatory, excess neoplasms or excess transport in either 2020 or 2021. In a stepwise regression of Covidother 2020, no variable entered the model at the 10% level of significance. In 2020, mean SI 2020 was positively (r=0.31, p=0.0888) associated with Covidother. Covidother was negatively correlated in 2021 with mean vaccination rate (r=-0.42, p=0.0142), and max vaccination rate (r=-0.46, p=0.0077) and was not correlated with stringency statistics but all were slightly negative. Covidother in 2021 was not correlated with stringency 2020 statistics.

There were no significant correlations between Covid-19 not identified (CNID) and Covid-19 other (CO) and excess mortality rates.

Part 2. Detailed results on the regression models that fitted cause-specific mortality rates to vaccination rates, economic, health, demographic and government response stringency index variables.

Overall excess and non-Covid-19 excess mortality

In the stepwise regression analysis in 2020 the HDI (p=0.04) and age 70 or older (p=0.05) were negative and median age positively (p=0.01) associated with excess mortality (R^2^=0.48) and in 2021 female smokers (p=0.02) and the SI mean (p=0.08) were positively associated and HDI (p<0.0001), life expectancy (p<0.0001) and max vaccination rate (p=0.0001) were negatively associated (R^2^=0.95).

Excess respiratory mortality

In the stepwise regression analysis of excess respiratory mortality in 2020, HDI (p<0.001) was negatively, and hospital beds positively (p=0.05) related (R^2^=0.57). In 2021 HDI (p=0.003), max vaccination rate (p=0.005) and median age (p=0.02) were negatively associated while the sd vaccination rate was borderline negatively associated (p=0.06) and R^2^=0.70. Note excess respiratory rates were negatively correlated with mean vaccination rate (-0.61, p=0.0002), sd vaccination rate (-0.56, p=0.0008) and max vaccination rate (-0.54, p=0.0013). Excess respiratory rates were also negatively correlated with the mean SI in both 2020 and 2021 but not significantly.

Excess circulatory mortality.

In the stepwise regression analysis in 2020, HDI was negatively (p<0.001) associated with excess circulatory (R^2^=0.45) and in 2021 the mean vaccination rate (p<0.001) and HDI (p=0.003) were negatively associated (R^2^=0.75). Note excess circulatory mortality was positively correlated with the mean, sd and max stringency index in 2020 and positively with the mean and negatively with sd and max in 2021 but not significantly.

Excess neoplasms mortality.

In a stepwise regression in 2020, gdp (p=0.002) was negatively associated with excess neoplasms (R^2^=0.30) and in 2021 male smokers (p<0.001) and HDI (p=0.005) were both positively associated (R^2^=0.35). However, when Cyprus was omitted, male smokers was no longer significant in the model and age 70 and older (p<0.001) and female smokers (p<0.05) were positively associated, and median age negatively associated (p<0.01), with R^2^=0.52. Correlations between excess neoplasms in 2020 with the SI in 2020 and 2021 statistics were all very small and negative. Excess neoplasms in 2021 was negatively correlated with min vaccination rate (-0.37, p=0.036).

Excess transport accidents mortality.

In a stepwise regression of excess transport 2020, HDI (p<0.001) and sd SI (p=0.05) were positively associated, population density (p=0.04), female smokers (p=0.04) and life expectancy (p=0.04) negatively associated (R^2^=0.58). In 2021 age 70 older (p=0.0159), HDI (p=0.0194) was positively associated while population density (p=0.001) was negatively associated (R^2^=0.51). Population density was negatively associated with excess transport both years. This was driven by Malta with a population density almost three times any other country and with a low excess transport mortality rate. When Malta was omitted population density was not significant. All correlations of excess transport with stringency statistics were negative but not significant in 2020 and 2021. Excess transport 2021 was not significantly correlated with vaccination statistics and all correlations were very small. Excess transport in 2021 was not significantly correlated with stringency 2020 statistics.

Excess other non-Covid mortality

In a stepwise regression of excess other 2020, no variable entered the model at the 10% level of significance. In 2021 excess other was negatively associated with HDI (p=0.014) and positively associated with excess respiratory in 2021 (p=0.005). All correlations of excess other with stringency statistics were not significant in 2020 and 2021. Excess other was positively correlated with vaccination statistics in 2020 and negatively in 2021 (apart from min vaccination rate in 2021), significantly with maximum vaccination rate in 2021 (p=0.037) but all correlations were small. Excess other in 2021 was not significantly correlated with stringency or vaccination statistics in 2020.

Reference.

**Beeks VV, et al*.*** (2024) Cause-Specific excess mortality during the COVID-19 pandemic (2020–2021) in 12 countries of the C-MOR consortium. *Journal of Epidemiology and Global Health;* **14**: 337–348. <https://doi.org/10.1007/s44197-024-00242-4>

Figure Legend

Figure S1. Mortality rates standardised per 100,000 of the population for “other” and non-Covid causes pre-Covid and 2021 with lines of identity. Pre-Covid rates are the averages for 2016-2019. Countries are labelled by their ISO code.

Supplementary Table S1: Covid-19 (identified CID, otherwise Covidoth) standardised mortality rates per 100,000 population in 32 European countries in 2020 and 33 in 2021 †.

| Country | CID2020 | Covidoth2020 | CID2021 | Covidoth2021 |
| --- | --- | --- | --- | --- |
| Austria | 68.46 | 2.93 | 83.51 | 2.04 |
| Belgium | 140.46 | 41.02 | 70.50 | 13.49 |
| Bulgaria | 105.37 | 8.40 | 365.17 | 8.37 |
| Croatia | 107.13 | 0.29 | 203.07 | 0.95 |
| Cyprus | 19.45 | 0.00 | 86.89 | 0.26 |
| Czechia | 108.11 | 1.81 | 242.68 | 10.85 |
| Denmark | 18.9 | 0.40 | 25.48 | 0.43 |
| Estonia | 14.31 | 0.14 | 125.61 | 0.80 |
| Finland | 8.66 | 0.50 | 15.17 | 0.22 |
| France | 74.99 | 11.76 | 76.21 | 1.07 |
| Germany | 38.95 | 1.28 | 71.42 | 1.70 |
| Greece | 38.73 | 0.06 | 127.08 | 0.12 |
| Hungary | 94.35 | 1.85 | 255.6 | 2.59 |
| Iceland | 11.51 | 0.40 | 1.70 | 0.00 |
| Ireland | 53.32 | 1.62 | 84.87 | 0.36 |
| Italy | 94.77 | 5.95 | 81.95 | 0.57 |
| Latvia | 34.57 | 0.19 | 214.71 | 0.65 |
| Liechtenstein | 130.32 | 0.00 | 52.63 | 4.46 |
| Lithuania | 76.07 | 0.14 | 236.79 | 0.55 |
| Luxembourg | 95.1 | 4.45 | 83.21 | 2.77 |
| Malta | 43.57 | 0.00 | 43.89 | 0.00 |
| Netherlands | 108.1 | 16.64 | 116.18 | 1.84 |
| Norway | 8.68 | 0.04 | 16.9 | 0.72 |
| Poland | 116.95 | 4.19 | 255.73 | 11.68 |
| Portugal | 58.33 | 0.94 | 102.58 | 0.23 |
| Romania | 90.80 | 1.23 | 222.52 | 0.22 |
| Serbia | 124.44 | 20.79 | 381.16 | 18.85 |
| Slovakia | 86.98 | 0.00 | 314.74 | 2.16 |
| Slovenia | 161.73 | 0.00 | 133.1 | 7.17 |
| Spain | 112.19 | 25.82 | 73.26 | 1.18 |
| Sweden | 85.85 | 4.05 | 49.18 | 0.47 |
| Switzerland | 104.04 | 1.90 | 66.34 | 0.77 |
| Turkey | N/A | N/A | 156.98 | 0.00 |
| † *, **, *** denotes statistical significance at the 0.05, 0.01 and 0.001 level respectively. | | | | |
