# Supplementary figures and images for: Excess respiratory, circulatory, neoplasm, and other mortality rates during the Covid-19 pandemic in the EU and their implications
Source: Epidemiol Infect. 2025 Jul 14;153:e86. doi: 10.1017/S0950268825100265 (PMC12322785; doi:10.1017/S0950268825100265)

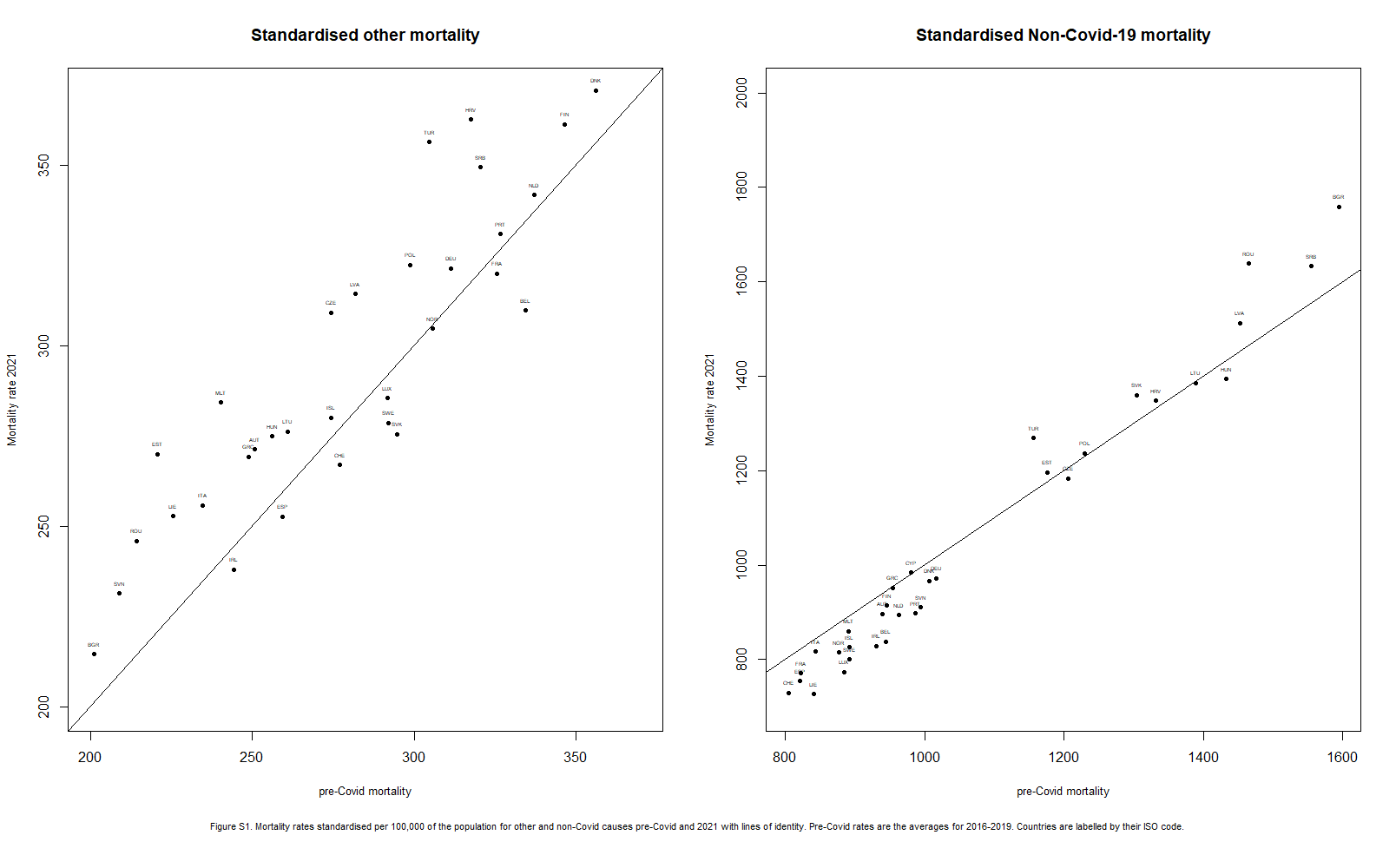

Supplement: Kelly et al. supplementary material [file S0950268825100265sup001.zip › GkellyRevisionFigure S1.tiff]
